# Conceptualising Climate Vulnerability in Health Sciences Through Scenario-Based Learning

**DOI:** 10.3390/ijerph23040439

**Published:** 2026-03-31

**Authors:** Manfred Fiedler, Daniela Schmitz

**Affiliations:** Department for Human Medicine, Innovative and Digital Methods of Teaching and Learning in Multiprofessional Health Care, Witten/Herdecke University, D-58455 Witten, Germany

**Keywords:** climate change, vulnerability, climate events, ecological public health, higher education, scenario-based learning

## Abstract

**Highlights:**

**Public health relevance—How does this work relate to a public health issue?**

- Climate events have threatening health impacts on social groups, which are temporarily, specifically, or generally vulnerable.
- Dealing with vulnerability to climate events leads to a special approach, which can be described as ecological public health.

**Public health significance—Why is this work of significance to public health?**

- For health professionals, it is of great importance to understand the fundamental aspects of climate change and the resulting impacts on health in order to prepare for and respond to different critical climate events.
- Critical climate events can therefore be understood as public health emergencies. They always have spatial effects and thus need a public health response, which has to include concepts of developing climate competencies for health professionals. This work focuses on the implementation of teaching and learning concepts in higher education as part of primary and additional education.

**Public health implications—What are the key implications or messages for practitioners, policy makers, and/or researchers in public health?**

- While the effects of the altered planetary processes on regional climatic conditions are becoming apparent, professional climate awareness and climate competences are, on the one hand, fundamental prerequisites for contributing to the development and implementation of health policy measures to protect people and communities against the health effects of climate events within the framework of municipal climate preparedness and, on the other, to develop personal resilience to cope with these events in everyday professional life.
- In terms of research, it is important to establish evidence whether the early inclusion of climate-related teaching in primary qualifications and through interdisciplinary or interprofessional teaching and learning concepts, such as problem-based learning or scenario-based learning, can be successful.

**Abstract:**

Since anthropogenic climate change is one of the most serious future challenges and therefore a major risk for public health, it is a crucial aspect of public health. Therefore, ecological public health has become a novel paradigm within public health, which mainly focuses on the concept of climate vulnerability. One of the most important questions is how health professionals should be prepared for handling different climate events in their professional field. Knowledge about climate events and their impact on health, especially of vulnerable groups, is crucial. The article discusses the integration of climate change in healthcare education using the example of two teaching and learning projects that we conducted. While it is hard to train on climate events in practice, we discuss scenario-based learning as a teaching–learning method using two case studies: in a multiprofessional course feed and in a multidisciplinary student group. We evaluated the outcome by different methods. As an additional evaluation tool, we used a questionnaire so that students could reflect on their learning experiences. Second, we conducted group discussions as part of the learning and teaching concept to reflect on the learning outcomes. We found that scenario-based learning is a suitable teaching–learning method for supporting professional awareness about the impacts of climate change on human health, depending on the learning environment and the structure of the learning group.

## 1. Introduction

Climate change and human interventions in planetary processes have serious effects on human health, with considerable consequences for human health. Therefore, they create new demands for health professionals regarding qualifications and professional perspectives on how to act on climate events and the long-standing health effects of climate change. The aim of this article is, on the one hand, to discuss the resulting scientific impacts on higher education in health sciences. On the other hand, we discuss teaching and learning concepts that deal with climate vulnerability. Critical climate events as a spatial outcome of climate change with serious impacts on human health cannot be trained in practice, so we introduced scenario-based learning as a crucial teaching and learning format. We want to reflect on the experiences gained from two learning projects in terms of the students’ learning experiences, learning objectives, and learning outcomes. On this basis, we will assess, by way of example, whether and to what extent teaching and learning concepts can contribute to professional climate awareness and climate competence.

## 2. Climate Change as a Future Hazard to Human Health

Humans, until now, are the only species on earth that have the mental and social competencies to permanently transform the surface of this planet. This kind of transformation historically began with the advent of agriculture and, with it, the enduring settlement of humans and the constitution of villages and cities [1]. Since industrialization began at the end of the 18th century, anthropogenic interventions have become of a comprehensive dimension that induces a critical influence on planetary processes and therefore on planetary stability [2,3]. The loss of biodiversity, the change of atmospheric processes, and the rise of average global temperature due to an increase in the share of atmospheric greenhouse gases, foremost carbon dioxide, methane, and dinitrogen monoxide, are severe consequences that the world and human civilization are facing [4,5].

The last decades have seen a considerable global increase in the number and intensity of climate-related events, i.e., heatwaves, droughts, thunderstorms, wildfires, or flooding. In Europe, the yearly days of high temperatures (above 30 °C) have tripled in the last 30 years [6]. While the number of wildfires seems to be merely constant, the burnt area per wildfire has increased massively. For example, 19 of the 20th largest wildfires in California occurred in the last 25 years [7]. Thunderstorms and heavy rainfall have become more intense and are a threat in all parts of the world. Whilst in low- and middle-income countries the loss of life and livelihood is the main threat, in high-income countries, high economic losses are the main consequences [8,9,10].

Most of these events have serious implications for human health. People with certain capabilities, i.e., age, chronic diseases, or social disadvantages, are most threatened by climate events. For example, the regional rise in average temperatures has serious effects on the elderly and people with chronic diseases, such as diabetes mellitus, coronary heart disease, or pulmonary impairment [11]. Flooding threatens people with cognitive decline and people with impairments [12]. Droughts, flooding, and thunderstorms have an increasing influence on agricultural crops and might raise consumer prices for basic foodstuffs, which is a serious threat to people with a subsistence income, especially for people in low-income countries [13,14].

## 3. Ecological Public Health

In times of climate change, ecological and environmental conditions are becoming crucial aspects of people’s health. Therefore, in addition to social determinants of health (SDH) [15], we can identify ecological determinants of health (EDH) as one major issue for health and health promotion. While SDH is focusing on the relationship between health and the social conditions of people and individuals, EDH prioritizes the embedment of humans in the ecosystem and the natural and social environment they are living in and from [16]. There are substantial overlaps between SDH and EDH, especially with regard to environmental resources of people and the interconnection to people’s social resources.

As one of the forefathers of EPH, McMichael [17] makes the distinction between a steady-state point of view on public health and the perspective of public health in a world in change, especially due to the increase in atmospheric greenhouse gases. Based on their analysis, Rayner & Lang [18] developed an understanding of EPH as an integrated perspective of ecological epidemiology as a tool to assess the complexity of comprehensive health influencing factors now and in the future. Thus, EPH is a social group-based approach, based on a tablet of delocalised sources, focusing on environmental threats for human health, as there are “rapid, massive population growth; climate change; resource scarcity and price volatility; soil, sea and freshwater damage; biodiversity loss” [19] speaks of the disease triangle, consisting of interconnected social system processes, ecological system processes, and the individual’s conditions. Coutts et al. refer to the embedment of the human built environment in the ecosphere and “the complex reciprocity” [20] between the natural environment and human activities.

So, EPH seems to be a novel paradigm that extends public health issues by integrating questions of planetary processes and their sustainable protection as a matter of health promotion. Probably because of the complexity of the relationship between ecological change, human interventions, and the effects on people’s health, the ascertainment of what ecological public health methods appear similar to is not well elaborated. Traditionally, public health has focused on characteristics of social groups to identify health threats as a result of social environments, cultural aspects, or behavior, using means of biomedical interventions and behavioral change [21]. In recent times, social and spatial interventions have been added to ensure the effectiveness of health-promoting programs [22,23,24]. Ecologically oriented interventions enlarge the professional and disciplinary field of reference. Interventions have to be addressed that are not typically associated with social care and with health care, but might have effects on the health of social groups or the municipal or local population. An important tool for action on ecological health hazards, therefore, should be a broad understanding of vulnerability to ecological hazards and its assessment.

## 4. The Concept of Climate Vulnerability

Due to the complexity of the impacts of climate events on regions, systems, organisations, and individuals, climate vulnerability is to be understood as a holistic, multidimensional concept of how people are influenced by climate change and the competencies to cope with it. Therefore, a comprehensive definition of climate vulnerability includes different perspectives on vulnerability against climate events, whether it is a systems perspective [25], a spatial focus on topography and built environment [26], or the institutional approach, especially for health care facilities [27]; any of these influences the well-being of communities, social groups, and individuals.

While vulnerability on the personal level depends on the capabilities of the individual or the social group, systems, spatial, or organisational vulnerability is not a steady-state type of definition of vulnerability. It focuses on different factors that could have impacts in the sense of creating more or less resistance or resilience against climate events over time [28].

The assessment of climate vulnerability is three-fold: risk assessment on the one hand, the assessment of sensitivity on the other hand, and third, the evaluation of the dependence of humans on the functional integrity of social and natural systems, organisations, and communities and their stability facing climate events [29].

Especially the lives of people with severe chronic illnesses often depend on so-called critical infrastructure [30]. For example, floods or wildfires might temporarily disable the electrical supply in affected areas, which could have a critical effect on people with home ventilation [31].

Natural disasters are not new in the history of mankind and have happened before anthropogenic climate change. But due to rising global average temperatures as a result of an increasing share of atmospheric greenhouse gases, climate events, as single or slow-onset disasters, have been increasing in number and/or in severity in the last fifty years [32].

## 5. Climate Vulnerability and Health Care Education

### 5.1. Education on Climate Vulnerability

Thus, climate vulnerability as a scientific concern requires multidisciplinary perspectives, while in the practical field of interventions on climate vulnerability, there is a need for multi- or interprofessional collaboration. Although there is increasing interest in education on health and climate change and knowledge about climate events and personal vulnerability, a comprehensive educational concept on behalf of a multidimensional perspective on climate vulnerability and its implications for ecological public health is still missing.

Methods of interdisciplinary teaching and learning and interprofessional education have been more established in recent times in patterns of higher education [33]. Especially, the pattern of climate change, its health impacts, climate vulnerability, and climate adaptation have become an issue of greater interest [34]. However, climate vulnerability is an additional issue because curricular restrictions make it hard to integrate it into primary and higher education in health sciences [35]. For example, in Germany, in human medicine, a field-oriented modularisation has recently been recommended by the German Science Council [36], but the body of teaching facilities is still oriented at the medical subdivisions with a high degree of specialisation [37,38]. It is even more difficult to integrate pluridisciplinary content into the curricula or even to create interdisciplinary student groups of thematically referenced disciplines.

### 5.2. Climate Education

Nevertheless, several aspects of teaching and learning concepts can be identified in the literature. One of the main focuses lies on the distinction between theories and topics within the field of educational content. From the perspective of the definition of climate competence, Fuertes et al. [39] defined a category-based framework as a tool to analyse the curricular insertion of climate change and health.

Two theoretical concepts seemed to be layers for the content of educational concepts on climate change, namely the 17 United Nations Goals of Sustainability and, second, the concept of the Planetary Health Framework. Both concepts, as integrated items in education focusing on the development of knowledge about the sources of climate change and the complex interconnection with climate change and societal and personal health, are therefore aimed at supporting climate competence in the professional practice field. With Sorensen et al. [40], we can describe climate competence by five domains: knowledge and analytical skills, interdisciplinary and interprofessional collaboration and communication, policy skills and understanding, public health practice, and clinical practice. Wiek et al. [41] identify key competencies to analyse and deal with complex problems on sustainability topics. These include system-thinking competencies, strategic competencies, interpersonal competencies, as well as normative competencies to deal with sustainability values, and anticipatory competencies to imagine future scenarios. The mitigation of climate change remains at the centre of the goals of this teaching and learning concept of climate education [42].

The focus is on so-called transformative learning in the sense of being enabled to change personal and organizational behavior in order to develop a path of decarbonizing daily routines and to install processes of sustainable professional practice. Transformative learning aims for profound changes in the thinking and behavior patterns of learners. Learners should be enabled to understand complex social challenges, to contribute to their solution, and to critically reflect on their own assumptions and convictions. According to Mezirow [43], learners transform their taken-for-granted frames of reference, such as meaning perspectives, habits of mind, and mindsets. Transformation learning means to reflect on these frames and to re-frame them referring to knowledge gain, new experiences, and communication with others in order to become mindful and “clear thinking decision makers” [41], (p.7f). For health professions, this means knowing about the impact of professional practice on climate change and developing competencies to find solutions for a sustainable transformation of the professional field of practice.

A more far-reaching educational approach is focusing on the impact of climate change and climate events on health, especially for vulnerable persons and social groups. This leads to the question of how health care professionals are prepared to professionally handle occurring climate events, such as wildfires, thunderstorms, heat waves, or flooding. Facing an increase in critical climate events in recent years, advanced education, for example, in disaster medicine or disaster nursing as a specialisation to manage natural disasters, has become more crucial. In this kind of climate education, the main learning goal is to prepare health care professionals for managing critical climate events as natural disasters with a large number of affected persons, i.e., mass casualties, in and with potentially critical, partially life-threatening conditions, which may afford triage and critical short-term decisions [44,45]. The last is an example of the importance of reflecting aspects of ethical decision-making within climate education [46].

### 5.3. New Perspectives on Climate Education

Since the COVID-19 pandemic, the term ‘slow onset disaster’ or ‘slow onset event’ has been introduced into the understanding of disaster management [47,48]. This term helps to identify some impacts of climate change, such as events that influence communities’ and individuals’ health over a longer period with more or less intensity over time.

This broader understanding of natural disasters is mostly relevant for health professionals and medical and health education. One fundamental should be, first, the reference to an eco-social approach to public health education for the task of climate prevention action on health, and second, a systematic pathology of climate-related impacts on health and sickness. This means nothing less than building professional competence for the assessment of individual climate-related health risks. This is not only relevant in inpatient care, especially from the perspective of emergency or critical care, but even more so with the focus on chronic health conditions in residential and outpatient care.

Therefore, issues of climate impacts can be introduced in primary education for health professions as a curricular enhanced modular-based training on specific chronic or acute health conditions. The eco-social approach allows for the specification of social and natural environmental conditions that influence climate vulnerability and vice versa, the climate resilience of individuals and social groups. Basically, it is critical to generate ‘climate science literacy’ that enables health professionals to manage critical climate events using science-based evidence in their everyday practice [49].

In educational practice, approved teaching and learning concepts include outdoor teaching and learning [50], problem-based learning, case studies, and scenario-based learning. Especially in advanced education, for example, for residential doctors, digital or online learning concepts have been developed with the aim to quickly introduce climate knowledge into everyday practice and support the climate literacy of health professionals [51,52].

Despite the importance of climate change for health, there is still no appropriate path available for the introduction of climate health as a regular issue in health science education, especially with regard to primary education.

## 6. Scenario-Based Learning as a Teaching-and-Learning Method on Climate Vulnerability

### 6.1. Scenario-Based Learning

Scenario-based learning as a teaching-and-learning method is most suitable for learning tasks when training in practice is not possible due to the nature of the learning object. Errington [53] defines scenario-based learning as an educational approach with intentional use of scenarios with situated knowledge bound to the context to achieve learning objectives in a near-world context. Scenarios can thus vary from straightforward sets of circumstances and conditions to complex event sequences that incorporate storylines, roles, and team dynamics, allowing students to navigate through multiple pathways and resulting in a wide range of possible outcomes [54]. Didactic concepts with near-world scenarios provide learners with relevant and authentic learning opportunities, which connect to professional practice beyond specialized knowledge [53].

In this paper, we examine two different learning environments where we used scenario-based learning as a teaching and learning method.

### 6.2. First Case Proceeding

In the first case, we introduced scenarios as a simulation game in a multi-professional learning group as part of a degree program in the field of dementia and chronic care (*n* = 19). The task of students was to find solutions to support elderly people in urban areas in case of a heat wave. Two student groups worked on self-specified scenarios and developed solutions based on the specific scenario within two months. In the first step, each group elaborates its scenario to work on, for example, the characteristics of the quarter (density of residential development, infrastructure, or the focused groups of elderly individuals with specific chronic conditions). In the second step, each student group assessed health risks and vulnerabilities regarding their scenario. Then, they developed a solution that may be suitable for protecting the elderly from health risks during a heatwave. Finally, a peer discussion organized as a competitive arena to evaluate the solutions in the student group was conducted. The student groups presented their solutions, discussed them within the peer group, which determined the solution and how it should be put into practice. At the end of the process, we led a focus group discussion [55] to reflect on the learning process as such. The opening question was how the students experienced the process of elaborating public health solutions facing the hazard of heat events. The academic teachers organised the group discussion and summarised the results in writing, after the student group consented to the main outcomes of the discussion.

### 6.3. First Case Results

The results of this methodological approach yielded some interesting answers regarding the use of scenario-based learning in the field of climate vulnerability. First of all, heat events, as an important outcome of climate change, were identified as a wicked problem. The students felt a rising awareness about it by using the method of scenario-based learning, especially as a simulation game. As a result, they perceive a growth in action competence to find solutions for such problems. Collaboration with other professions leads to the development of transdisciplinary competencies, which are suitable for dealing with complex problems in practice. On the other hand, they realize (multiprofessional) networking as effortful. Thus, they find it difficult to manage and coordinate the implementation of found problem solutions in practice.

Another problem is that teaching-and-learning methods are very often not a normal part of the curriculum. The simulation game, especially with the task to plan the implementation of solutions in real life, led to a high workload in addition to the mandatory parts of the degree course.

### 6.4. Second Case Proceeding

In the second case, we introduced scenario-based learning as a crucial teaching-and-learning method for a multidisciplinary seminar dealing with climate vulnerability. The learning goal of the seminar was to introduce the super-complexity of climate change and its impacts on the health of people and societies into the mono-disciplinary perspective of students from different study courses. It was crucial to find common ground to develop competencies to act professionally on climate change.

The seminar consisted of 14 students from three different primary qualification courses in health sciences, namely psychology, human medicine, and dentistry. It was conducted for the first time. So, the initial aim of the teaching project was to find out whether the concept of introducing the problem of climate vulnerability in a course of different health disciplines is suitable for making it relevant for action in practice.

The learning-teaching concept of the seminar aims to increase students’ understanding and reflection on the mechanisms of climate change and on how it influences professional practice in health care and the instruments to respond.

At the beginning, it was important to create a joint understanding of the mechanisms of climate change and its impact on health, and the vulnerability of communities and social groups exposed to different climate events. These inputs of joint knowledge about climate change should enable the students to discuss and reflect on instruments to respond to climate events.

Therefore, three teaching–learning methods had been introduced in the seminar:

- Peer-to-peer reflection and discussion, which gave the opportunity to establish common ground and therefore understanding about the problem field.
- Case studies: We introduced real climate events. Mixed student groups identified the proposed solutions, discussed them, and probably found more appropriate ways to cope with climate events.
- Scenario-based learning: At the end of the seminar, we designed scenario-based learning activities, where the students should discuss the possible effects of health events. They should work on complex interventions to prepare for the focused climate event at a community level from the perspective of certain health professionals.

This proceeding had the learning goal to strengthen awareness about the impacts of climate change and climate events on health and the resulting challenges for health professionals and their professional practice.

We evaluated the conceptualisation of the seminar and the didactical proceeding by two methods. First, we used a questionnaire at the beginning and at the end of the seminar as an extended evaluation tool for the seminar. Unlike the usual instrument for evaluating seminar quality, it gave us the opportunity to measure changes in perspective on interprofessional practice and awareness about the impact of climate change on professional practice. The questionnaire consisted of questions about attitudes towards climate change and health on the one hand, and questions about experiences of interdisciplinary learning related to the subject of the seminar on the other. The questionnaire used a five-point Likert scale to measure changes in attitudes towards climate change resulting from the seminar.

For this article, we just analysed the questions on climate attitude. The questions focused on the learning goals of the seminar; we tested the results with a *t*-test, which is suitable for small sample sizes (see Table 1):

We compared the changes in attitudes between the start and the end of the seminar. Significant changes have been highlighted in the table.

Second, in the last session of the seminar, we conducted a focus group discussion with all participating students [55], which allowed us to gain additional insight into the seminar experiences from a personal and a joint perspective. While filling in the questionnaire was voluntary, the focus group discussion was an integrated part of the seminar.

The group discussion was structured around three initial questions:

- How do we argue the scientific evidence of climate change and health?
- What is the impact of climate change on me as a health professional and as a personally affected person?
- What are the most important insights from learning for my future practice?

As part of the seminar concept, the discussion followed a student-centred approach. The above questions were the only prerequisites for the discussion. The content and results were determined by the students. To encourage every student to participate, each student was initially asked to make a statement about the initial questions. The results of the discussion were recorded by the teacher with open reassurance from the students.

### 6.5. Second Case Results

The main results were somehow astonishing, and at least in some aspects, not expected:

1. In higher education, the focus of teaching and learning lies on the scientific aspects of a problem field. The scientific significance of climate change was initially recognised by the students. After the seminar, it was considered by the students to be of even more importance. The teaching and learning concept, with the final scenario-based approach, strengthened the scientific competence to reflect on a complex scientific object. It was the outcome of the discussion and the result of the questionnaire.
2. On the other hand, as a result of the discussion, reflecting on the complexity of the topic in a seminar, which was conducted weekly over a short period of three months, led to cognitive overload. This means that the students felt overwhelmed by the scientifically complex content and the multitude of phenomena to deal with.
3. While the seminar focused on climate vulnerability, the effects on health, especially on vulnerable individuals and groups, were considered an important insight.

Nevertheless, the significance of the professional field in healthcare was considered less important after the seminar sessions. At first glance, this seems contradictory. But on the one hand, the excessive demands in terms of content seem to lead to a cautious attitude towards practical implementation. On the other hand, the discussion pointed to the requirements of the healthcare system. From the students’ perspective, these determine the possibility and ability to act professionally. This is also reflected in the results of the questionnaire. Although the willingness to become personally involved has increased significantly overall, including at work, there have been no significant changes in terms of how discussions at work are perceived.

4. Participatory formats, especially peer-to-peer formats as an interdisciplinary form of collaboration, facilitate access to problem perception and problem-solving tools. Case studies and, finally, joint scenario-based learning support competencies in problem-solving in the field of climate vulnerability.
5. However, the transfer of knowledge into practice, in the sense of transformative action competence with a view to professional practice, could not be assessed, nor has it improved following the results of the questionnaire. Students stated in the discussion that they did not feel able to transfer the solutions they had developed or reflected on to their own future professional fields of activity.
6. In conclusion, not all learning objectives were achieved. Climate knowledge and awareness of the complex impacts were strengthened. On the other hand, transformative thinking and action competence for practice cannot yet be addressed.

## 7. Discussion

While in the first case, the student group had been studying together for some time (two years) and had developed a distinct common ground in the forefield, they could therefore more easily choose a common interdisciplinary approach to the subject area of climate vulnerability. The teaching and learning concept in the second case is based on an interdisciplinary approach, where the seminar was conducted in addition to the regular course schedule. Students and teachers had to develop a common ground during the seminar sessions. The students mainly so far have not had multidisciplinary experiences. Moreover, interdisciplinary learning is not a self-running process; it must first be learned. This includes establishing a shared knowledge base and dealing with challenges such as communication, differing object descriptions, methods, and workflows in the spirit of productive irritation [56].

Another aspect should be recognised: the students in the first case were mature students, while in the second case, the majority of students had been first-year students. So, what can be identified as a professional or disciplinary identity might not have been well elaborated. With Wyatt et al. [57], the taking of responsibility correlates with having gained a professional identity. Therefore, as an academic teacher, it is crucial to know about the linkage of responsibility to professional identity, in this case, with regard to climate change. It might be important to discuss it at the beginning of the climate education process.

Grounding complex problems requires a distinct competence, which is sometimes called interdisciplinary or transdisciplinary competence [58]. Basically, a solid foundation with the knowledge base and the basic methodology of one’s own discipline is an important recommendation for working with multidisciplinary content and for being enabled to engage in interdisciplinary reflection and finding solutions [59]. Thus, the effectiveness of teaching and learning might improve for the more experienced students with the study in the main discipline.

As a major result of both cases, awareness of climate change and vulnerability can be strengthened, which is common with other findings [60,61]. In scenario-based learning, generic inputs are made as a preliminary for the students, so that they can shape it individually or as a student group [54]. Accordingly, creating scenarios is a method that allows students of various disciplines and different professional experiences to advance in education by working with complex problems based on individual perspectives and their joint capabilities.

Since climate change and climate vulnerability are not an integrated part of the curricula of academic courses, especially in primarily qualifying degree programs, academic teachers have to ascertain the capabilities of the students in the learning group, especially facing the mixed structure of learning experiences. As a voluntary part of the degree program, it can be assumed that students’ participation is based on high motivation for the subject of the seminar. However, the complexity of climate change as a subject of study means that students with different academic and personal backgrounds can become overwhelmed despite their high level of motivation. Scenario-based learning, as a student-centred teaching and learning method, does indeed facilitate easier personal access [62]. However, in our cases, both groups of students were borderline stressed, at least quantitatively, and in the second case, also qualitatively.

The advantages of this teaching–learning concept for professional practice seem to be evident. However, our results show that on the one hand, the requirements for the mostly extraordinary interventions should not be underestimated, which means that when realising found solutions into practice, it cannot be taken for granted that it fits with the daily professional requirements. Thus, it is not self-evident that experiences in a problem-focused teaching–learning concept by applying near-world scenarios could easily be connected to future professional practice. One of the important tasks of academic teachers is to present scenarios that are understandable to students and at the same time relevant to practice [63].

All in all, the results of the two small inquiries support the usage of scenario-based learning under certain preconditions, depending on the learning environment, i.e., first of all, the integration into the degree program and the structure of the learning group.

## 8. Limitations

There are some limitations: First of all, the number of students participating was limited due to the course format. So, the findings from the two teaching and learning projects cannot be generalised. We consider this to be a study of specific cases because of the specialty of the teaching-and-learning form in the first group and the initial establishment of the course format in the second group. Thus, results cannot easily be compared. Further research on teaching and learning concepts is necessary. The multidisciplinary course concept could not be observed over a longer period. It should be considered whether a different alignment of the course can help to resolve issues arising in the project relating to unachieved learning objectives. It is also worth discussing whether age, prior qualifications, the stage of study (e.g., master’s or bachelor’s degree, or the stage of medical studies), have an influence on the results. It was not the subject of this study; data on this were therefore not requested from the students.

## 9. Conclusions

Scenario-based learning is a suitable teaching and learning method for highly complex problems in healthcare. Real-world scenarios based on real experiences with climate events could provide a common basis for students and healthcare professionals to find solutions for dealing with them. However, the gap between the teaching environment and the practical spatial environment should not be underestimated. For both students and healthcare professionals, dealing with issues relating to the health effects of climate change represents an additional task alongside their normal practice. Furthermore, an increase in transformative competence in the field of professional practice cannot be taken for granted, particularly from the perspective of basic training.

In addition to issues of cognitive coping, this is not least due to structural reasons, which are an important task of public health.

Thus, the three basic principles of Mccabe et al. [64] on public health emergency preparedness, ability, willingness, and readiness, may be helpful. One task of higher education is to ensure that health professionals have the ability to respond to climate events. Public willingness takes the form of individual and collective climate awareness that recognises the significance of the effects of climate events on health and creates the conditions for coping with them. Finally, readiness means making resources available to implement measures when necessary.

Our experiences from the two teaching and learning projects show that problem-based teaching and learning methods are helpful when they refer to scenarios relevant to practice. However, in terms of the organisation of the degree courses, resources and free spaces are needed to make the complex problem area of climate and health manageable for students. In addition, practical aspects of healthcare and public health need to be incorporated in the form of a shared climate awareness to which university teaching and learning results can refer.

It is also important that the experience gained from such projects is used for their further development, not least because the aforementioned changes cannot be expected to be implemented in the short term. For example, the seminar concept should be modified so that it can achieve greater connectivity for students in their respective subject areas, thereby improving interdisciplinary exchange for better learning outcomes.

Due to the limitations of these case studies, more research on the topic is needed. The importance of demographic factors, such as the age of the students, but also existing academic and non-academic qualifications, and the students’ stages of study, appears to have an influence. This should be taken into account in future evaluations of teaching and learning methods in the field of climate change and health, and climate vulnerability. The knowledge-related prerequisites in the field of climate adaptation and climate vulnerability should also be examined, as they improve the outcome of scenario-based learning in this subject area. Finally, it seems sensible to conduct longitudinal studies for long-term teaching and learning formats.

## Figures and Tables

**Table 1:** Results and testing questionnaire.

Question	A2	Dif.	SD2	T	P
Environmental Knowledge Relevance	4.80	0.20	0.45	2.68	0.055
Solving climate change is a waste of time	1.20	0.00	0.45	0.00	1
Solving environmental problems without much changes	2.80	1.00	1.30	4.60	0.01
High professional contribution for solving climate crisis	2.60	0.00	0.89	0.00	1
High personal contribution for solving climate crisis	3.80	0.20	0.45	2.68	0.055
Relevance climate my current/futurep field of work.	3.60	−0.40	1.52	−1.58	0.189
Discussion Impact of climate change in the private sphere	3.90	0.10	0.22	2.68	0.055
Discussion Impact of climate change in the professional sphere	3.00	0.40	1.00	2.40	0.074
Discussion solution of climate change in the professional sphere	2.40	0.20	0.89	1.34	0.251
Personal engagement to solve problems of climate change in the professional sphere	2.60	0.40	0.55	4.38	0.012
Personal engagement to solve problems of climate change in the professional sphere	2.80	−0.40	1.10	−2.19	0.094

## Data Availability

The public release of raw data was not part of the informed consent process. The informed consent included the analysis of the qualitative data to evaluate the application of the method and its usefulness for multiprofessional education and thus for the development of teaching and learning. The data presented in this study are available on request from the corresponding author, as they contain sensitive content that could be misunderstood or misused if published openly.
